# Serological and molecular diagnosis of *Trypanosoma vivax* on buffalos (*Bubalus bubalis*) and their ectoparasites in the lowlands of Maranhão, Brazil

**DOI:** 10.1590/S1984-29612024066

**Published:** 2024-10-28

**Authors:** Thais Bastos Rocha Serra, Andrea Teles dos Reis, Carla Fernanda do Carmo Silva, Raynara Fernanda Silva Soares, Simone de Jesus Fernandes, Luiz Ricardo Gonçalves, Andrea Pereira da Costa, Rosangela Zacarias Machado, Rita de Maria Seabra Nogueira

**Affiliations:** 1 Programa de Pós-graduação em Ciência Animal – PPGCA, Universidade Estadual do Maranhão – UEMA, São Luís, MA, Brasil; 2 Imunodot Diagnósticos Veterinários – IMUNODOT, Jaboticabal, SP, Brasil; 3 Laboratório de Imunoparasitologia, Departamento de Patologia, Reprodução e Saúde Única, Faculdade de Ciências Agrárias e Veterinárias – FCAV, Universidade Estadual Paulista – UNESP, Jaboticabal, SP, Brasil

**Keywords:** Buffalos, Haematopinus tuberculatus, iELISA, Trypanosoma vivax, immunochromatographic test, Bubalinos, Haematopinus tuberculatus, iELISA, Trypanosoma vivax, teste imunocromatográfico

## Abstract

The aim of this study was to detect trypomastigote forms of *Trypanosoma vivax*, in blood smears, DNA of *T. vivax* and anti-*T. vivax* antibodies in samples from buffalos reared in the lowlands of Maranhão, Brazil. Blood samples were collected from 116 buffalos and 25 ectoparasite specimens. Blood smears were produced to diagnose forms compatible with *Trypanosoma* spp.; the indirect enzyme-linked immunosorbent assay (iELISA) and lateral-flow immunochromatography (Imunotest®) serological tests were used; and the polymerase chain reaction (PCR) was used to make molecular diagnoses. No forms compatible with *Trypanosoma* spp. were observed in blood smears. Among the 116 serum samples analyzed, 79.31% and 76.72% were positive in the ELISA and rapid tests, respectively. One sample was positive in the molecular test. Twenty-five lice of the species *Haematopinus tuberculatus* were collected. When subjected to PCR for detection of DNA of *T. vivax*, all of them were negative. The louse specimens were negative for *T. vivax*. There were no statistically significant differences (p < 0.05) in the presence of *T. vivax* in this region, in relation to the animals’ age and sex. It can be concluded that these protozoa are circulating in the buffalo herd of the lowlands of Maranhão displaying crypitc parasitemias.

## Resumo

O objetivo deste estudo foi detectar formas tripomastigotas de *Trypanosoma vivax* em esfregaço sanguíneo, DNA de *T. vivax* e anticorpos anti-*T. vivax,* em amostras de búfalos, criados na Baixada Maranhense, Brasil. Foram coletadas amostras de sangue de 116 búfalos e 25 espécimes de ectoparasitas. Para o diagnóstico, foi realizada a confecção de esfregaços sanguíneos a fim de observar formas compatíveis com *Trypanosoma* spp., a sorologia por Ensaio Imunoenzimático Indireto ((iELISA), a Imunocromatografia de fluxo lateral (Imunotest®) e o diagnóstico molecular por meio da Reação em Cadeia da Polimerase (PCR). Não foram observadas formas compatíveis com *Trypanosoma* spp. nos esfregaços sanguíneos. Dentre as 116 amostras de soro analisadas, 79,31% e 76,72% mostraram-se positivas nos ensaios de ELISA e teste rápido, respectivamente. Uma amostra foi positiva no teste molecular. Foram coletados 25 piolhos da espécie *Haematopinus tuberculatus.* Quando submetidos a PCR para a detecção do DNA de *T. vivax*, todos mostraram-se negativos. Os espécimes de piolhos estavam negativos para *T. vivax*. Não houve diferença estatística significante (p<0,05) em relação à idade e sexo dos animais e a presença de *T. vivax* na região. Pode-se concluir que o protozoário está circulando em rebanho bubalino da Baixada Maranhense, demostrando parasitemias crípticas.

## Introduction

Buffalos (*Bubalus bubalis*) were brought to Brazil in the 19^th^ century and became adapted to this country, notably in its northern region, where the climate and vegetation favored buffalo breeding. The anatomical and physiological hardiness of this species has made these animals more resistant to illnesses that are common in cattle (Bernardes, 2007; Damé, 2019). The zootechnical qualities of this species have boosted local livestock-rearing activities because of the value placed on its products and byproducts (i.e. milk, milk derivatives and meat), given their high-quality nutritional characteristics. Buffalo meat is considered to be relatively more healthy than bovine meat (Borquis et al., 2013; Simões da Silva et al., 2020).

The presence of *T. vivax* in buffalos was recorded in Brazil for the first time by Shaw & Lainson (1972), through blood smears obtained from animals on the island of Marajó, state of Pará. Subsequently, this parasite was detected in buffalos in the state of Amapá (Serra-Freire, 1981). However, it was only in 1995 that this parasite was first identified outside of the northern region of Brazil, when it was found in the Poconé region of the Pantanal, in the state of Mato Grosso (Silva et al., 1996).

Over the last few years, there were reports of this parasite in several Brazilian states: Mato Grosso (Silva et al., 1996), Mato Grosso do Sul (Paiva et al., 1997; Dávila et al., 2003; Ramos et al., 2020), Maranhão (Guerra et al., 2008; Pereira et al., 2018), Tocantins (Linhares et al., 2006), Paraíba (Batista et al., 2007), Minas Gerais (Carvalho et al., 2008), Rio Grande do Sul (Silva et al., 2009), Pernambuco (Pimentel et al., 2012), São Paulo (Cadioli et al., 2012), Alagoas (Andrade et al., 2019), Santa Catarina (Fávero et al., 2016), Goiás (Bastos et al., 2017), Sergipe (Vieira et al., 2017), Piauí (Lopes et al., 2018) and Pará (Dyonisio et al., 2021).

Flies of the genus *Glossina* are responsible for biological transmission of *T. vivax* in Africa, but mechanical transmission can also occur. However, *Glossina* spp. are absent from Central and South America and, consequently, *T. vivax* has become adapted to mechanical transmission in these regions. Its main vectors here are the dipterans *Tabanus* spp., but other vectors have also been reported as mechanical transmission agents, such as the dipteran species *Stomoxys calcitrans* and *Haematobia irritans* (Bastos et al., 2017; Cadioli et al., 2012). Lice of the species *Haematopinus tuberculatus* and ticks of the species *Rhipicephalus* (*Boophilus*) *microplus* and *Amblyomma cajennense* have been found to be positive for *T. vivax*, but little is known about the importance and involvement of these ectoparasites in the life cycle of *T. vivax* (Bolivar, 2013; Dyonisio et al., 2021). Iatrogenic transmission through sharing of contaminated surgical materials, syringes and needles at the time of applying medications and/or vaccines has also been reported (Batista et al., 2012; Bastos et al., 2017).

Trading and transportation of animals between states within a single country or between neighboring countries have facilitated the introduction of asymptomatic animals into new areas that had been considered to be free from *T. vivax*. This had led to the need to identify this parasite in areas where it has not yet been studied (Silva et al., 1998; Jones & Dávila, 2001; Cadioli et al., 2012).

Thus, the objective of this study was to detect trypomastigote forms of *T. vivax* in blood smears, DNA of *T. vivax* and anti-*T. vivax* antibodies through blood samples from buffalos reared in municipalities in the lowland region of Maranhão, Brazil.

## Material and Methods

### Study area

**Figure 1:**
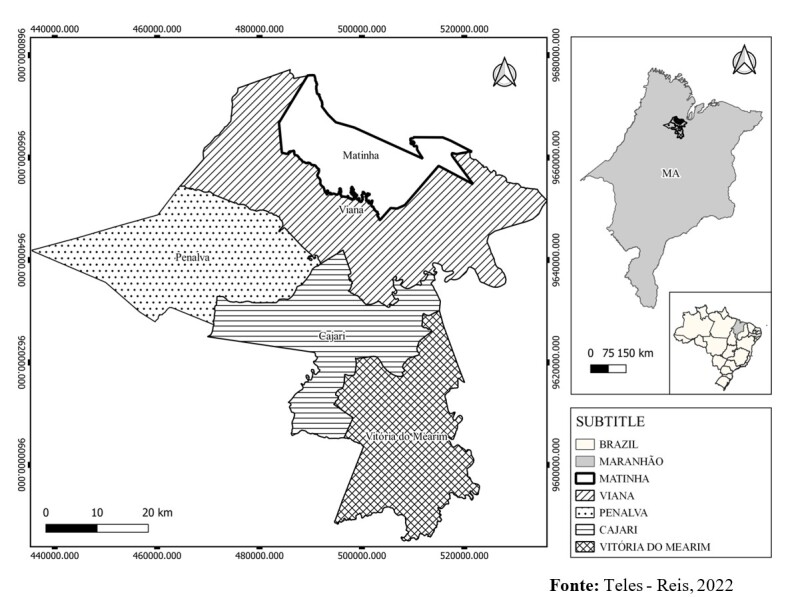
Map of the municipalities included in this investigation.

This investigation was conducted in five municipalities in the lowland region of the state of Maranhão: Vitória do Mearim, Cajari, Viana, Matinha and Penalva (Figure 1). This region lies within the northern mesoregion of Maranhão, delimited to the north by the coastline (Atlantic Ocean), to the south by the Cocais region, to the west by the pre-Amazon region and to the east by the Cerrado region. It is located between the coordinates 01°59” to 04°00” S and 44°00” to 45°33” W, and it covers a total area of 17,579.366 km^2^ (IBGE, 2021). The region is formed by a wide diversity of rich ecosystems that includes rivers, lakes, estuaries and areas subject to flooding, and it lies within the Brazilian Legal Amazon zone (IBGE, 2021).

### Animals

The animals sampled were selected randomly according to convenience, depending on the number of animals available at the time of the visit to the municipality. A total of 116 buffalos were included in this study (36 males and 80 females), distributed as follows: 20 animals in the municipality of Viana, 25 in Cajari, 30 in Vitória do Mearim, 24 in Matinha and 17 in Penalva. They were divided into three age groups: up to 11 months (< 12 months; one to three years (1-3 years); and over three years (> 3 years). All of the animals were of mixed breed, and their age range was from 4 months to 144 months. These animals were reared under extensive a semi-extensive system.

At the time of the visit to each farm, the following data were collected: identification of each animal, age, sex and ectoparasites.

### Obtaining and processing of samples

Capillary peripheral blood samples were obtained from the base of the tail or from ear vessels, to produce blood smears.

In addition, 5 ml of blood was collected by puncturing the jugular vein using the Vacutainer® system. The blood was collected both in tubes containing the anticoagulant ethylenediaminetetraacetic acid (EDTA) and in tubes without anticoagulant. The samples collected in tubes without anticoagulant were kept at rest for removal of the coagulum and then centrifugation at 2,000 G for 10 minutes. The resultant serum was transferred to Eppendorf tubes for serological analysis. The blood samples with EDTA were used for DNA extraction and for performing the polymerase chain reaction (PCR).

At the time of collecting the blood, the animals were inspected visually to collect ectoparasites. When detected, these were stored in 70% alcohol for subsequent identification in accordance with the dichotomous key described by Serra-Freire & Mello (2006), and for molecular tests to be performed.

### Blood smears

The blood smears were produced immediately after obtaining the blood. They were fixed using methanol and stained with Giemsa (2:30 for 15 minutes). They were then examined under an optical microscope at magnifications of up to 1000x, to investigate any presence of blood parasites.

### Indirect enzyme-linked immunosorbent assay (iELISA)

Serological diagnoses were made using the indirect enzyme-linked immunosorbent assay (iELISA), in accordance with the method described by Machado et al. (1997) and Aquino et al. (1999), with small modifications. Flat-bottomed Nunc MaxiSorp® plates (Thermo Fisher Scientific, Massachusetts, USA) were used. These were sensitized with 1 μg/mL of recombinant antigen of *T. vivax*, diluted in a carbonate-bicarbonate buffer (pH 9.6). The control and tested serum samples were diluted in the proportions 1:100 for *T. vivax*. Anti-bovine IgG alkaline phosphatase conjugate (Sigma-Aldrich®, A0705-0.25ML, Saint Louis, USA) was used, diluted in PBS Tween 20 in the proportions of 1:30,000. The cutoff point definition was according to Castilho et al. (2021).

The negative controls came from a female Holstein cow and a calf belonging to a herd located in region that is not endemic for trypanosomosis. These animals had previously undergone molecular testing (PCR) and serological testing (ELISA) for *T. vivax* infection, with negative results. The positive controls were selected from two cows that had been experimentally infected with *T. vivax*, which had been confirmed through PCR and ELISA.

The plates were read using an ELISA reader (B.T. -100; Embrabio, São Paulo, Brazil), with a 405 nm filter. The cutoff point was calculated as 2.5 times the average absorbance of the negative control serum samples (Machado et al., 1997).

### Lateral-flow immunochromatography technique (Imunotest®)

To detect specific antibodies against the recombinant protein of *T. vivax*, a lateral-glow immunochromatography test (rapid test) was also performed, according to the manufacturer’s recommendations (Imunodot Diagnósticos®, Jaboticabal – SP, Brazil).

### Molecular methods

#### Extraction of genomic DNA

DNA was extracted from whole-blood samples in EDTA, using InstaGene Matrix® (BIORAD), according to the manufacturer`s recommendations. DNA was also extracted from the ectoparasites using the BIO GENE kit (Bioclin). Extracted DNA was subjected to spectrophotometry analysis (UV/Vis spectrophotometer; Biodrop µLite). Subsequently, the material was stored at -20 ºC for molecular tests (PCR) to be performed.

#### Molecular diagnosis of *T. vivax*

The blood samples and individual ectoparasites were tested for *T. vivax* using PCR technique (Cortez et al., 2009). The PCR was performed in accordance with the technique described by Cortez et al. (2009), with the following oligonucleotide primers: TviCatL [5´ GCCATCGCCAAGTACCTCGCCGA3´] and DT0155 [5´ TTAAAGCTTCCACGAGTTCTTGATGATCCAGTA3´] (IDT®, USA). These amplified a fragment of 177 base pairs of the CatL-like gene of the catalytic domain region, which codes for the enzyme Cathepsin L-like (CatL) of *T. vivax*.

The reaction products were subjected to electrophoresis on 1.5% agarose gel in a horizontal bath with the buffer tris-acetate-EDTA (TAE) at 50 V/100 mA, and Syber Safe staining (Invitrogen). The amplified products were viewed under ultraviolet light in a transilluminator. Samples that presented bands at the level of the positive control were considered positive.

#### Purification and sequencing

The DNA fragments amplified in the molecular tests were purified using the Clean-Up system kit (Promega), in accordance with the manufacturer’s instructions. The products were subjected to sequencing in the Technology Department of the Center for Biological Resources and Genomic Biology (CREBIO), School of Agrarian and Veterinary Sciences (FCAV), State University of São Paulo (UNESP). The chain-terminating dideoxynucleotide method (Sanger et al., 1977) was used, in an ABI Prism 3700 DNA Analyzer sequencer (Applied Biosystems, Foster City, CA, USA).

#### Sequence analysis and phylogenetic inferences

Consensus sequences were obtained through analysis on electropherograms using the Phred-Phrap software (Ewing et al., 1998). The Phred quality score (peaks around each base call) was established as ≥ 20 (99% precision of base calls). The alignment of the *T. vivax* sequences was constructed and manually edited using the BioEdit software (version 7.0.2.5) (Hall, 1999). Phylogenetic analysis was performed using the maximum likelihood (ML) and neighbor-joining (NJ) methods. Phylogenetic trees were compiled using the MEGA-X software. The Akaike information criterion (AIC), available within MEGA-X (Tamura et al., 2011), was applied to identify the most suitable model for nucleotide substitution. The two-parameter Kimura model was chosen as the most appropriate for the phylogenetic analysis on the alignment of the *T. vivax* sequences.

### Statistical analysis

The absolute and relative frequencies of the pathogens identified in the serological and molecular tests were established. The analyses were performed through contingency tables using different variables according to Fisher’s exact test and the independent chi-square test, with the aid of the Epi Info™ software, version 7.2. The results were confirmed through the BioEstat 5.0 software.

Associations between variables and the frequency of seropositive findings were estimated in terms of a significance level of 5% (p < 0.05) and odds ratios (OR) with 95% confidence intervals.

## Results and Discussion

No trypomastigote forms of protozoa compatible with *Trypanosoma* spp. were viewed in the blood smears. This direct parasitological evaluation is very specific but has low sensitivity, especially in the chronic phase of the disease. It has higher sensitivity in acute cases or at the initial stages of the disease, when the peak level of parasitemia is generally associated with a febrile state (Radostits et al., 2002; Almeida et al., 2019). The animals evaluated in the present study did not present any apparent clinical symptoms, which would explain the absence of these parasites in the blood smears.

The serological tests for trypanosomosis due to *T. vivax* detected that a high percentage of the buffalos in the herds were seropositive: out of the total number of animals, 79.31% (92/116) had anti-*T. vivax* antibodies according to iELISA, with optical density above the cutoff (0.343 and 0.305). Moreover, 76.72% (89/116) of the samples showed positive reactions in the immunochromatographic test (Imunotest®). Through evaluating these results together, it was observed that only three animals that were seropositive in the iELISA test did not show a reaction in the immunochromatographic test. This level of detection of anti-*T. vivax* antibodies indicates that these animals were frequently in contact with the parasite, even if the level of parasitemia was so low that it could not be detected in blood smears. The parasitemia still had the capacity to induce formation of antibodies that could remain at detectable levels for a prolonged period (Cadioli et al., 2012; Fidelis et al., 2019).

In cattle herds in the states of Minas Gerais and São Paulo, Alcindo et al. (2022) and Cadioli et al. (2012) observed that high percentages of the animals were seropositive for *T. vivax*. The iELISA diagnostic method has high sensitivity, particularly among naturally infected animals (Castilho et al., 2021), and is a widely-used method for making this diagnosis in herds.

No statistically significant difference (p < 0.05) in the presence of antibodies against *T. vivax* was found between these age groups. Among cattle, it is already clear that age can be considered to be an aggravating factor for *T. vivax* infection, such that older animals are generally more susceptible and present greater occurrence than younger animals (< 12 months) (Dayo et al., 2010; Adam et al., 2012; Takeet et al., 2013; Angwech et al., 2015).

There was no statistically significant difference (p < 0.05) in the presence of antibodies against *T. vivax* between male and female buffalos. Likewise, in previous reports, there was no difference in the prevalence of *T. vivax* infection in buffalo herds according to the animals’ sex (Fikru et al., 2012; Biryomumaisho et al., 2013; Takeet et al., 2013).

**Figure 2:**
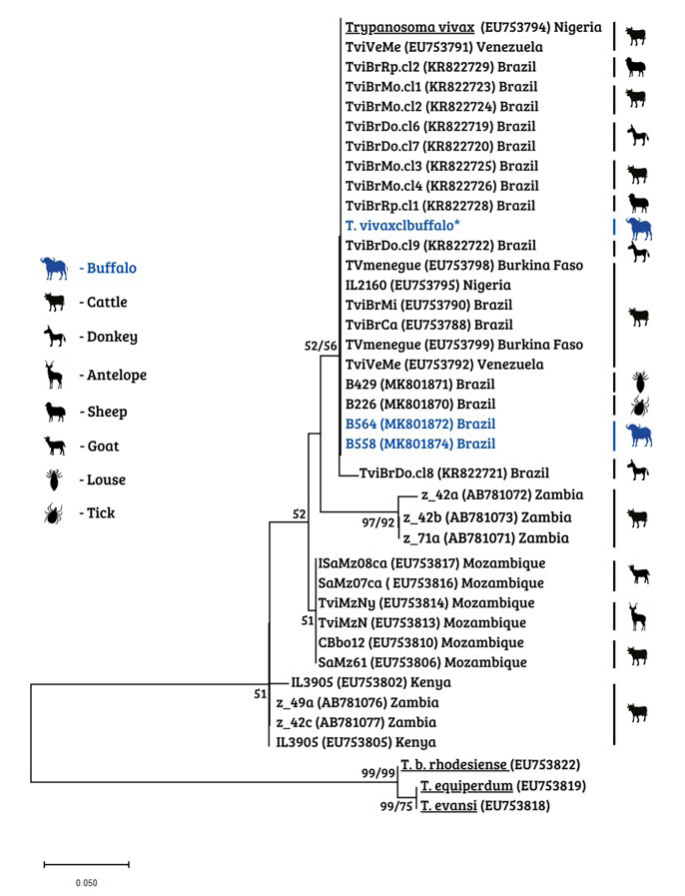
Phylogenetic relationships between sequences of *Trypanosoma vivax* based on the cathepsin L-like gene (CatL). This tree was inferred using the maximum likelihood and neighbor-joining methods, with the K2P model. The sequences detected in buffalos are in blue, and the amplification of the present study is marked with an asterisk (*). The numbers at the nodes correspond to bootstrap values greater than 50% that were accessed with 1,000 replications. *Trypanosoma brucei rhodesiense*, *Trypanosoma equiperdum* and *Trypanosoma evansi* were used as an outgroup.

In addition to serological methods, the samples were subjected to molecular detection, through which one animal was found to be positive for the parasite. The sequence of the PCR-amplified product from this positive sample has been deposited in GenBank with the accession code OR339796. The phylogenetic analysis confirmed the close relationship between the genotype of this sample that was positive for *T. vivax* and those of another two sequences of *T. vivax* in buffalos that were detected previously in Brazil and deposited in GenBank (MK801872 and MK801874) (Figure 2).

The buffalo that was detected as positive for *T. vivax* through molecular methods also showed a positive reaction through the serological techniques used in this study. Similar findings through using molecular techniques were reported by Dyonisio et al. (2021) among buffalos in the state of Pará and by Garcia et al. (2005) among buffalos in Venezuela and Argentina.

Records of the presence of *T. vivax* in Maranhão had previously been reported by Guerra et al. (2008), in a bovine calf in the municipality of Itapecuru-Mirim, which is located in the central-western region of this state, a municipality with characteristics differing from those of the lowlands of Maranhão. Pereira et al. (2018) reported the occurrence of an outbreak of disease caused by *T. vivax* in the state of Maranhão, but in cattle. The present report provides the first record of infection by *T. vivax* in buffaloes in this state.

At the time of inspecting the buffalos, 25 specimens of lice identified as *Haematopinus tuberculatus* were collected from 16 of these animals. This ectoparasite is considered to be specific for buffalos. The specimens of *H. tuberculatus* that were sampled were negative for *T. vivax*. Nonetheless, Dyonisio et al. (2021) reported occurrences of *T. vivax* in *Amblyomma cajennense, Rhipicephalus microplus* and *H. tuberculatus* in the state of Pará. However, there are no scientific evidences that these ectoparasites can transmit the parasite.

In conversations with buffalo farmers, they reported that no quarantine was implemented when new animals were introduced to the herd. This is a matter that ought to be considered, in order to prevent dissemination of *T. vivax* to regions where this disease agent is not present. Moreover, the lack of veterinary supervision at the time of applying medications to buffalos means that needle-sharing, a practice that has been reported to be a mechanical form of transmission of *T. vivax*, remains possible.

## Conclusions

In the present study, we report that there was high seroprevalence of *T. vivax* among the buffalos sampled in the lowland region of Maranhão. On the other hand, out of the 116 animals sampled, only one animal was found to be positive through PCR. There was no significant difference in seroprevalence in relation to the variables of the age or sex of the animals. These animals could contribute towards dissemination of buffalo trypanosomosis in the region, in case of stressful factor, such as co-infections that leads to increase in parasitemia. This report provides the first record of infection by *T. vivax* in buffalos in the state of Maranhão.

## References

[B001] Adam Y, Marcotty T, Cecchi G, Mahama CI, Solano P, Bengaly Z (2012). Bovine trypanosomosis in the Upper West Region of Ghana: entomological, parasitological and serological cross-sectional surveys. Res Vet Sci.

[B002] Alcindo JF, Vieira MCG, Rocha TVP, Cardinot CB, Deschk M, Amaral GG (2022). Evaluation of techniques for diagnosis of *Trypanosoma vivax* infections in naturally infected cattle in the Zona da Mata Mineira. Rev Bras Parasitol Vet.

[B003] Almeida LS, Sena LM, Barioni G, Moraes TMA, Oliveira RE (2019). Comparação entre métodos de avaliação direta para o diagnóstico de babesiose em bovinos. Res Soc Dev.

[B004] Andrade AQ, Mendonça CL, Souto RJC, Sampaio PH, Fidelis OL, André MR (2019). Diagnostic, clinical and epidemiological aspects of dairy cows naturally infected by *Trypanosoma vivax* in the states of Pernambuco and Alagoas, Brazil. Rev Bras Med Vet.

[B005] Angwech H, Nyeko JHP, Opiyo EA, Okello-Onen J, Opiro R, Echodu R (2015). Heterogeneity in the prevalence and intensity of bovine trypanosomiasis in the districts of Amuru and Nwoya, Northern Uganda. BMC Vet Res.

[B006] Aquino LPCT, Machado RZ, Alessi AC, Marques LC, Castro MB, Malheiros EB (1999). Clinical, parasitological and immunological aspects of experimental infection with *Trypanosoma evansi* in dogs. Mem Inst Oswaldo Cruz.

[B007] Bastos TSA, Faria AM, Madrid DMC, Bessa LC, Linhares GFC, Fidelis OT (2017). First outbreak and subsequent cases of *Trypanosoma vivax* in the state of Goiás, Brazil. Rev Bras Parasitol Vet.

[B008] Batista JS, Riet-Correa F, Teixeira MMG, Madruga CR, Simões SDV, Maia TF (2007). Trypanosomiasis by *Trypanosoma vivax* in cattle in the Brazilian semiarid: description of an outbreak and lesions in the nervous system. Vet Parasitol.

[B009] Batista JS, Rodrigues CMF, Olinda RG, Silva TMF, Vale RG, Câmara ACL (2012). Highly debilitating natural *Trypanosoma vivax* infections in Brazilian calves: epidemiology, pathology, and probable transplacental transmission. Parasitol Res.

[B010] Bernardes O (2007). Bubalinocultura no Brasil: situação e importância econômica. Rev Bras Reprod Anim.

[B011] Biryomumaisho S, Rwakishaya E-K, Melville SE, Cailleau A, Lubega GW (2013). Livestock trypanosomosis in Uganda: parasite heterogeneity and anaemia status of naturally infected cattle, goats and pigs. Parasitol Res.

[B012] Bolivar AM (2013). Detección de *Anaplasma marginale* y *Trypanosoma vivax* en garrapatas de ganado bovino empleando la reaccion en cadena de la polimerasa. Rev Electrón Vet.

[B013] Borquis RRA, Araujo FR, Baldi F, Hurtado-Lugo H, Camargo GMF, Muñoz-Berrocal M (2013). Multiple-trait random regression models for the estimation of genetic parameters for milk, fat, and protein yield in buffaloes. J Dairy Sci.

[B014] Cadioli FA, Barnabé PA, Machado RZ, Teixeira MCA, André MR, Sampaio PH (2012). First report of *Trypanosoma vivax* outbreak in dairy cattle in São Paulo state, Brazil. Rev Bras Parasitol Vet.

[B015] Carvalho AU, Abrão DC, Facury EJ, Paes PRO, Ribeiro MFB (2008). Ocorrência de *Trypanosoma vivax* no estado de Minas Gerais. Arq Bras Med Vet Zootec.

[B016] Castilho KJGA, Garcia ABCF, Fidelis OL, Nagata WB, André MR, Teixeira MMG (2021). Follow-up of dairy cattle naturally infected by *Trypanosoma vivax* after treatment with isometamidium chloride. Rev Bras Parasitol Vet.

[B017] Cortez AP, Rodrigues AC, Garcia AH, Neves L, Batista JS, Bengaly Z (2009). Cathepsin L-like genes of *Trypanosoma vivax* from Africa and south America – characterization, relationships and diagnostic implications. Mol Cell Probes.

[B018] Damé MCF (2019). Sanidade de bubalinos no Extremo Sul do Brasil..

[B019] Dávila AMR, Herrera HM, Schlebinger T, Souza SS, Traub-Cseko YM (2003). Using PCR for unraveling the cryptic epizootiology of livestock trypanosomosis in the Pantanal, Brazil. Vet Parasitol.

[B020] Dayo GK, Bengaly Z, Messad S, Bucheton B, Sidibe I, Cene B (2010). Prevalence and incidence of bovine trypanosomosis in an agro-pastoral area of southwestern Burkina Faso. Res Vet Sci.

[B021] Dyonisio GHS, Batista HR, Silva RE, Freitas e Azevedo RC, Costa JOJ, Manhães IBO (2021). Molecular Diagnosis and Prevalence of *Trypanosoma vivax* (Trypanosomatida: Trypanosomatidae) in Buffaloes and Ectoparasites in the Brazilian Amazon Region. J Med Entomol.

[B022] Ewing B, Hillier L, Wendl MC, Green P (1998). Base-calling of automated sequencer traces using *Phred*. I. Accuracy assessment. Genome Res.

[B023] Fávero JF, Da Silva AS, Biazus AH, Volpato A (2016). *Trypanosoma vivax* infection in goat in west of Santa Catarina state, Brazil. Comp Clin Pathol.

[B024] Fidelis OL, Sampaio PH, Gonçalves LR, André MR, Machado RZ, Wijffels G (2019). Comparison of conventional and molecular techniques for *Trypanosoma vivax* diagnosis in experimentally infected cattle. Rev Bras Parasitol Vet.

[B025] Fikru R, Goddeeris BM, Delespaux V, Moti Y, Tadesse A, Bekana M (2012). Widespread occurrence of *Trypanosoma vivax* in bovines of tsetse- as well as non-tsetse-infested regions of Ethiopia: a reason for concern?. Vet Parasitol.

[B026] Garcia H, Garcia M-E, Perez H, Mendoza-Leon A (2005). The detection and PCR-based characterization of the parasites causing trypanosomiais in water-buffalo herds in Venezuela. Ann Trop Med Parasitol.

[B027] Guerra RMSN, Feitosa AB, Santos HP, Abreu-Silva AL, Santos ACG (2008). Biometry of *Trypanosoma vivax* found in a calf in the state of Maranhão, Brazil. Cienc Rural.

[B028] Hall TA (1999). BioEdit: a user-friendly biological sequence alignment editor and analysis program for Windows 95/98/NT. Nucleic Acids Symp.

[B029] IBGE (2021). Pesquisa da pecuária municipal.

[B030] Jones TW, Dávila AMR (2001). *Trypanosoma vivax* - out of Africa. Trends Parasitol.

[B031] Linhares GFC, Dias FC, Fernandes PR, Duarte SC (2006). Tripanossomíase em bovinos no município de Formoso do Araguaia, Tocantins (relato de caso). Cienc Anim Bras.

[B032] Lopes STP, Prado BS, Martins GHC, Beserra HEA, Souza MAC, Evangelista L (2018). *Trypanosoma vivax* in Dairy Cattle. Acta Sci Vet.

[B033] Machado RZ, Montassier HJ, Pinto AA, Lemos EG, Machado MRF, Valadão IFF (1997). An enzyme-linked immunosorbent assay (ELISA) for the detection of antibodies against *Babesia bovis* in cattle. Vet Parasitol.

[B034] Paiva F, Lemos RAA, Oshiro AE, Salvador SC, Nakasato L (1997). Ocorrência de *Trypanosoma vivax* em bovinos no Estado de Mato Grosso do Sul. Rev Bras Parasitol Vet.

[B035] Pereira HD, Simões SVD, Souza FAL, Silveira JAG, Ribeiro MFB, Cadioli FA (2018). Aspectos clínicos, epidemiológicos e diagnóstico da infecção por *Trypanosoma vivax* em rebanho bovino no estado do Maranhão. Pesq Vet Bras.

[B036] Pimentel DS, Ramos CAN, Ramos RAN, Araújo FR, Borba ML, Faustino MAG (2012). First report and molecular characterization of *Trypanosoma vivax* in cattle from state of Pernambuco, Brazil. Vet Parasitol.

[B037] Radostits OM, Blood DC, Gay CC (2002). Clínica veterinária: um tratado de doenças dos bovinos, ovinos, suínos, caprinos e equinos..

[B038] Ramos IAS, Mello VVC, Mendes NS, Zanatto DCS, Campos JBV, Alves JVA (2020). Serological occurrence for tick-borne agents in beef cattle in the Brazilian Pantanal. Rev Bras Parasitol Vet.

[B039] Sanger F, Nicklen S, Coulson AR (1977). DNA sequencing with chain terminating inhibitors. Proc Natl Acad Sci USA.

[B040] Serra-Freire NM, Mello RP (2006). Entomologia & acarologia na medicina veterinária..

[B041] Serra-Freire NM (1981). Oiapoque: outro foco de *Trypanosoma vivax* no Brasil. Rev Bras Med Vet.

[B042] Shaw JJ, Lainson R (1972). *Trypanosoma vivax* in Brasil. Ann Trop Med Parasitol.

[B043] Silva AS, Costa MM, Polenz MF, Polenz CH, Teixeira MMG, Lopes STDA (2009). Primeiro registro de *Trypanosoma vivax* em bovinos no Estado do Rio Grande do Sul, Brasil. Cienc Rural.

[B044] Silva RAMS, Morales G, Eulert E, Montenegro A, Ybañez R (1998). Outbreaks of trypanosomosis due to *Trypanosoma vivax* in cattle in Bolivia. Vet Parasitol.

[B045] Silva RAMS, Silva JA, Schneider RC, Freitas J, Mesquita DP, Mesquita T (1996). Outbreak of trypanosomiasis due to *Trypanosoma vivax* (Ziemann, 1905) in bovine of the Pantanal Brazil. Mem Inst Oswaldo Cruz.

[B046] Simões da Silva TM, Piazentin ACM, Mendonça CMN, Converti A, Bogsan CSB, Mora D (2020). Buffalo milk increases viability and resistance of probiotic bacteria in dairy beverages under in vitro simulated gastrointestinal conditions. J Dairy Sci.

[B047] Takeet MI, Fagbemi BO, Donato M, Yakubu A, Rodulfo HE, Peters SO (2013). Molecular survey of pathogenic trypanosomes in naturally infected Nigerian cattle. Res Vet Sci.

[B048] Tamura K, Peterson D, Peterson N, Stecher G, Nei M, Kumar S (2011). MEGA5: molecular evolutionary genetics analysis using maximum likelihood, evolutionary distance, and maximum parsimony methods. Mol Biol Evol.

[B049] Vieira OLE, Macedo LO, Santos MAB, Silva JABA, Mendonça CL, Faustino MAG (2017). Detection and molecular characterization of *Trypanosoma* (*Dutonella*) *vivax* in dairy cattle in the state of Sergipe, northeastern Brazil. Rev Bras Parasitol Vet.

